# Hospital and Institutional News

**Published:** 1913-01-25

**Authors:** 


### HOSPITAL AND INSTITUTIONAL NEWS.

### THE FIRSTFRUITS OF PANEL SERVITUDE.

IN spite of the action of the British Medical Association in formally releasing its members from their pledge not to take service under the Insurance Act without its permission, the victory of the Chancellor —or should we say of the Insurance Commissioners?—is not so complete as it seems. There are widespread complaints, which are not likely to lessen in numbers, of the amount of clerical work which is imposed upon medical men on the panel. At an inquest held at Lambeth on Monday last the jury excused Dr. Hickson, a panel practitioner, who was called at an inquest on an insured man who died of strangulated hernia while having been given a prescription for colic, "owing to the scandalous amount of work imposed on him by the Insurance Act." Dr. Hickson said that he was so overwhelmed with the clerical work which fell to the lot of panel doctors that he had no time to examine the man. The Coroner, Mr. Ingleby Oddie, remarked that if the man had gone to a hospital his life would probably have been saved by an operation. One of the points about panel work can be best expressed in Dr. Hickson's own words: "People are not asking for treatment; they are simply asking to be put on the doctor's list." Again, when Dr. Robert S. Trevor, pathologist at St. George's Hospital, who had made a post-mortem examination, was called, the following dialogue occurred between him and Mr. Oddie:— The Coroner: "If this man had gone to the hospital he would have been alive now?"—"Probably." "As it is he went to his National Insurance Act doctor, and he is now dead?"—"Yes." "Do you think doctors ought always to examine a patient when there is a history of abdominal pain and sickness?"—"Personally I think certainly if it is possible." Without any attempt to fog the issue, this case seems to us a perfect illustration of the incidents which the Insurance panels, as they have been constructed, are likely to repeat in large numbers. But it does not explain Dr. Hickson's action in writing a certificate that death was due to enteritis when he had never examined the case. This is the old sort of sweated contract practice from which the poor have so long suffered; the Insurance Act was to have ushered in a brighter era, but the firstfruits are not refreshing, though we hope that they may be rare.

### MEDICAL VIEWS OF "PRO RATA" PAYMENTS.

IN a very just leading article on "National Insurance as it Affects the Voluntary Hospitals," *The Lancet* of last week pointed out that the profession had necessarily been so much absorbed in the medical benefit problems of the Insurance Act as scarcely yet to have had time to consider the bearings of the Act upon the hospitals. After summarising the attitude of the institutions as represented by the British Hospitals Association, the article proceeds to acknowledge in very kind terms the efforts which THE HOSPITAL has so persistently made to clarify the position, and to lay down a definite policy adequate to the difficulties of the case. The policy of *pro rata* payments, with an added 10 per cent. for the remuneration of the medical staffs is too comprehensive to command at once the assent or criticism of our professional contemporary, which, however, certainly takes the wise view in commending it to the attention of medical men. We should be less practised advocates than we are if we desired a hasty assent to these proposals. The institutions have been sufficiently impressed with their force, and now that the attention of medical men has been directed to them from a different quarter a wider sifting of discussion will follow, to the lasting good, as we believe, of hospitals and doctors alike.

### HAVE THE RED CROSS UNITS RETURNED TOO SOON?

IF any valid judgment may be based upon the individual feelings of some of those who as members of the Red Cross units returned last week from the war, their recall may yet prove to have been premature. This is not so much because a fear exists of a renewed outbreak of extended hostilities, but from the fact that hostilities have not yet virtually come to an end. Besides this, every war leaves an aftermath of disease the extent of which is never fully realised until the war itself is actually concluded. Last week the arrivals began. Dr. C. M. Page, resident surgeon at St. Thomas's Hospital, arrived with his party, including Dr. M. C. Gardner, Dr. E. E. Steele, and Dr. H. L. Mann; also Dr. Jennings, of St. Bartholomew's Hospital, who saw plenty of surgical work in the Stamboul Hospital. Dr. Steele arrived at Constantinople on November 3, and by the next evening the first patients arrived. His opinion on the question of the units' return would be interesting, for, though it is unnecessary to prophesy, many considerations suggest themselves why those units which were earliest upon the scene should be given a respite, and a voluntary organisation is always rather in a category apart.

### CHAPEL SERVICES IN A COMMITTEE ROOM.

THE temporary conversion of a recreation or committee room into a chapel for religious services on Sunday and at other times is still an event common enough to be regarded as a necessary *pis aller* in many institutions. The effect of such a compromise on the staff is too little considered. While, no doubt, many institutional superintendents accept it with a pious hope that a chapel may be built some day, others who take a less careless view have very definite opinions on the subject. While allowing at once that the chaplain is the proper person to control the services, the superintendent has a definite interest in the effect of the services upon his staff, if only for this reason. There is so little outlet for the emotions in the scheme of institutional life, that the wise superintendent is always anxious that the fullest results for good may accrue at all times of relaxation. Religious services are, or ought to be, one of the chief of such outlets, for many authorities entitled to respect hold strongly that the average worker finds his devotions impeded when they are publicly performed in a room of purely lay and humdrum association. From a committee room turned into a chapel the afflatus is lacking. It is easy to say that such ought not to be the case. But the fact that there is no religious body without its ceremonial—whether it be the studied bareness of white wall and prominent pulpit or the marble altar rich with fresco, lamps, and hangings—is sufficient to prove that the effect of atmosphere on worship has always been admitted. Who has not found, when no exciting cause has lent solemnity by contrast, that the Sunday services in a ship's dining saloon lacked the appeal of church or chapel at home? If that is generally admitted by people who are on a holiday, how much more will it be felt by institutional workers who need the afflatus of religion in greater degree by far? Should not, then, every institution, as a matter merely of first-rate justice and administrative importance, have its own chapel at all costs? We invite the experience of our readers on its value to the staff.

### THE PROBLEMS OF A COVERED WAY.

The total sum required for the reconstruction of the Children's Hospital, Leicester, including the cost of furnishing, is stated as £11,100, and an appeal has been made to the friends of the institution by the Chairman, Sir Edward Wood, for eighty-eight contributions of £100, so that the hospital might be opened free of debt. Allusion was made at a recent meeting to Sir Edward's seventy-fifth birthday, and the hope was expressed that he might be cheered with many evidences of good will in his present appeal. We notice also that there was a reference to the great need for a covered way between the infirmary and the new nurses' home, and, now that the wintry weather has demonstrated the need for it more acutely than before, the estimated cost of about £300 seems not to much to expect from some generous donor. It was indicated by the Chairman that the appointment of almoners was under consideration, and a scheme would shortly be presented to the board on this matter.

### AN UNFORESEEN DILEMMA.

Covered ways between an institution and a nurses' home raise sometimes more than one administrative problem. Where the buildings are new and the paths and lawns freshly laid, dry weather tempts the nurses from the covered way into the open, with the result that the new gravel works such havoc with their shoes as to make the institution's leather bill a heavy one. To avoid this human nature, even when in uniform, to judge by a recent case we have in mind, took to walking on the grass with disastrous results to the young lawns. In the end it became necessary to replace the gravel with asphalte, whereby both shoes and grass were saved in the dry weather.

### A WOMAN SECRETARY IN LONDON.

From time to time we have to chronicle the accession of a woman to the ranks of hospital secretaries. The latest instance appears to be that of Mrs. F. J. Pink, who succeeds her late husband as secretary of the Royal Dental Hospital. Perhaps, however, the change is almost as much nominal as real, since Mrs. Pink on many occasions supplemented those efforts which a long illness prevented her husband from exhibiting with regularity. It is noteworthy that the hospital is one kind of institution which has stood out, as it were, longer than many others as the province of male administrators. But the growing scope and importance of secretarial work is allowing a subdivision of responsibilty, in which perhaps women may become more in request.

### THE IRVINE CHAIR OF BACTERIOLOGY.

The intentions of the late Mr. Irvine, F.C.S., who died in 1902, with the curious and instructive provisions of his will, are now maturing. He left estate to accumulate in the hands of trustees till it reached the sum of £25,000 or £30,000, to be then devoted to founding the "Robert Irvine Chair of Bacteriology," the professor to be one of the faculties of medicine and surgery at Edinburgh University. The annual free income of the first sum is to be paid to the professor, and the income of the remaining £5,000 is to be spent on equipping the class-rooms and laboratories which will be associated with the Chair. A Parliamentary Paper was issued last Tuesday containing an Ordinance of the Court of the University. The residue of the estate is to be divided between the Royal Infirmary, the Royal Association for Incurables, and the Dunlop Cancer Fund at Edinburgh.

### THE VOLUNTARY HOSPITALS AND THE GOVERNMENT.

The discussion which took place at the meeting at Barnsley, when Lord Crewe spoke, which we reported fully last week, has aroused a good deal of interest. Several correspondents who were present have written their impressions, one hospital secretary writing that "he hugely enjoyed Sir Henry Burdett's speech following that of Lord Crewe at Barnsley." The vice-chairman of another hospital writes: "It was evident that Sir Henry Burdett carried the audience with him in favour of the voluntary system of working English hospitals. Lord Crewe, at great length, sought to prove that the Insurance Act would not injure the hospitals, but Sir Henry showed that in the near future greater strain would be placed upon them, and that, for some time at any rate, the hospitals would be less able than formerly to bear it. Sir Henry's suggestion of Government building loans to the hospitals and of Government payments *pro rata* for extra beds for insured patients met with a hearty reception." Another correspondent supplies this omission of the *Telegraph's* reporter: In the course of his speech "Sir Henry Burdett paid a tribute to Mr. Lloyd George's courage and sincerity in dealing with the matter of National Insurance, and thought they owed him much for trying to render better the lives and the working strength of the humblest poor in these islands. He further pointed out that the faults of the Act were due to unjustifiable haste, the prevention of all knowledgeable discussion upon its clauses, and a striking absence of statesmanship which permitted so complicated a measure to be placed on the statute book under such destructive conditions."

### A LEGAL POINT FOR PANEL DOCTORS.

An interesting point in legal medicine will probably arise about the end of this quarter, when the different public bodies, such as municipalities, borough councils, and the like, send out their payments for notifications of infectious diseases under the Public Health Act. Apart from tuberculosis, in regard to which a special arrangement is in force, there is a fee of half-a-crown payable for each notification by a private practitioner. But if the case has been notified by a doctor "in his practice as Medical Officer of a Public Body or Institution," then a fee of one shilling only is provided. If an Insurance Committee is a "public body"—and surely it must be—this may mean that panel doctors will be remunerated at the lower rate, instead of at the higher, for all notifications of infectious disease among insured persons. This presupposes that a panel doctor comes within the term "Medical Officer." The Financial Secretary to the Treasury thinks he is "not in the position of an employee of an Insurance Committee"; but it remains to be seen whether he is or is not a "Medical Officer." We are not aware that this point has been raised hitherto; but it is certainly worthy of consideration. In fairness we maintain that the half-crown fee ought to continue to prevail; and if it turns out that the wording of the Public Health Act, taken in conjunction with the provisions of the National Insurance Act, does not allow of it, steps should be taken to get the law altered at the earliest opportunity. At the same time it would be simple justice to abolish the shilling fee altogether; institutional medical officers are the greatest sufferers from this difference of scale, and we see no reason why they should continue to be deprived of that not extravagant remuneration for notifications which their colleagues in private practice enjoy. But unless someone takes the lead in the matter we doubt whether anything will be done. It rests with institutional officers themselves to ventilate this grievance, and the Insurance Act offers them a good opportunity.

### DEATH OF A MENTAL SUPERINTENDENT.

Dr. Joseph Bayley, who for forty-eight years has been medical superintendent of St. Andrew's Hospital for Mental Diseases at Northampton, died last Sunday at Llanfairfechan, in his seventy-ninth year. After an education at Reading School and King's College, London, he seized the opportunity offered by the Crimean War to volunteer for naval service. On his return, after having been present at the bombardment of Kronstadt, he became assistant medical officer at Leicester County Mental Hospital. Similar posts were also held by him at the Rutland, Salop, and Montgomeryshire County Mental Hospitals. Of the latter institution he became medical superintendent in succession to his father-in-law, Dr. Oliver. In 1865 he was appointed medical superintendent of St. Andrew's Hospital, an institution for upper and middle class patients which was established in 1838 and has a daily average of some 400 patients. In its own field the hospital has developed considerably in size since he was placed in charge, and it was at its branch at Llanfairfechan that he was taken ill when making his inspection in the autumn. Lunacy is the only field of work in which a man, however able, would be permitted to remain the responsible head of a great administrative establishment until his seventy-ninth year. Dr. Herbert Bayley, the eldest son of the late superintendent, has been senior assistant medical officer for some time past at St. Andrew's, where he has been for nearly twenty years. Dr. Herbert Bayley graduated M.D. at Edinburgh University in 1911.

### SALARIES OF MENTAL HOSPITAL WORKERS.

In our issue of January 4 reference was noted that the London County Council had recently increased the salaries of certain grades of workers at its mental hospitals. Some further concessions have now been made. At the Manor Mental Hospital maximum rates of remuneration for the clerk and house steward have been increased from £260 to £300 a year in the case of the clerk and from £255 to £300 a year in the case of the house steward. The rate of increase also has been increased in each case from £10 to £15 a year. At the Epileptic Colony the maximum rate of pay for the head attendant and assistant farm bailiff has been increased from 38s. to 40s. a week, to be reached by annual increments of 1s. a week. The maximum salary of the chief night watch has been increased from £70 to £75 a year, to be reached by two annual increments, one of £2 and one of £3.

### THE BRAVERY OF SCIENCE.

Attention has been deservedly drawn to the heroic decision of Dr. George Turner, who received a knighthood at the New Year, to devote his energies to the lepers, among whom, we fear, he must himself be counted. As we recorded at the time when Sir George's knighthood was announced, he was superintendent of the Pretoria Leper Asylum. Soon after his retirement two years ago he discovered, in his home in Devonshire, that he had contracted the disease. With the bravery of the man of science Sir George has looked the difficult facts in the face, and has determined to return to his former work. First, it is understood, he offered his services to the Mission to the Lepers in India and the East, but South Africa, where he has studied the disease and its conditions, seems to be preferred by his advisers. In the course of an interview with a lay newspaper representative Sir George is reported to have said that, though in his opinion leprosy is usually, if not always spread by contagion, most lepers are not nearly so dangerous to the public as a person suffering from pulmonary tuberculosis. If only such a point could be brought home to those panic-stricken ratepayers who treat every suggestion for the building of a sanatorium in their neighbourhood, as if it were on a par with the thrusting of another individual into an already overcrowded house, there would be some chance of restoring them to their common sense. Perhaps the best description of courage is past experience of victory over fear—a lesson badly needed by the panic-stricken public of one or two well-known cities, and magnificently illustrated by the life of Sir George Turner.

### MEDICAL MARTYRS: TRUE AND FALSE.

In a former issue of The Hospital we dealt in a leading article with the subject of medical martyrs in connection with the life-history of Semmelweiss. That aspect of the question, as therein adumbrated, is a sad one. Dr. Turner's work as Medical Officer of Health in the Transvaal brought him in contact with a disease which is prevalent in South Africa —with leprosy. In his official capacity he had to visit and work in the leper settlements in the Colony, and from the first his keen interest was aroused in this much discussed but comparatively little known disease. His laboratory work, carried on with painstaking care and that close attention to detail which characterise his investigations, was often done under adverse conditions. But the result has shown that in his case, as in that of some of his predecessors who have also attacked the problem of leprosy, precautionary measures are not always efficacious. We lay some stress on these points because Dr. Turner's case is one in which such admiration is wholly justifiable and even necessary. It is the duty of the profession to protest against the exaltation to the hierarchy of martyrs of medicine of those doctors who purchase a temporary glory in the lay press by sucking the diphtheritic membrane from a tracheotomy tube. Such "heroism" is merely un skilfulness, which needs condemnation, not praise. Nor is the public's admiration likely to be asked for scientists who inoculate themselves with pathogenic organisms to prove facts which are already well known, and which may be demonstrated by other and less drastic methods. Those who indulge in such investigations must bear themselves the blame for whatever results; all that we can give them is a modicum of the glory that is reflected on the captain who obstinately prefers to drown with his ship while there is a chance of safety without a loss of prestige. Every practitioner who has gone through his course at a large hospital can record cases of brilliant men on the staff or among his contemporaries, whose careers were cut short by their zealous efforts to do their whole duty to their patients, their students, and their institutions. A paragraph in the annual report, a laudatory notice in *The Lancet*, a regretful appreciative comment when their old fellow-students and pupils meet and the talk turns on the old days—these, for the most part, are all the honours they can claim. It is not, perhaps, that they look forward to greater glory. They know what they have taken in hand, and if some of them under-estimate the risk, the danger of the actuality soon undeceives them. But it is justifiable to ask whether the public, which benefits by what they have done, should altogether exclude them from admiration or appreciation, and whether the profession is right in hiding their honour under obituary notices which the layman never reads.

### A SCOTCH COTTAGE HOSPITAL MOVEMENT.

A Committee on Medical Service in the Highlands has now published its report, and its suggestion for dealing with the inevitable difficulties attaching to domiciliary treatment in the Highlands and Islands is the provision of cottage hospitals within comfortable reach of a doctor's residence. Midwifery patients, it is urged, would especially benefit by such provision. It is pointed out in this connection that the Belford Hospital, Lochaber, was provided expressely for "the wives of poor shepherds living at a distance and about to be confined." The cottage hospitals, in addition, would form, it is contended, a suitable residence for a district nurse, and would supplement the lack of consulting and dispensing accommodation in many of the remoter doctors' houses. The hospitals which at present exist present also the paradox of being few in number to the population and even so often half empty. The explanation given is not that the population is healthy, but that the management is bad. What do the authorities of the Gesto Hospital, Edinbane, Isle of Skye, say to this?

### A SUBSTITUTE FOR FUNERAL FLOWERS.

Funeral trappings are fortunately becoming less and less popular, but while the phrase "no flowers by request" is an increasingly common one in obituary announcements, suggested alternatives wear an air of novelty. Last week we drew attention to Mr. N. L. Cohen's death and to his connection with the London Hospital. To that connection may be added these words, which occurred in the official announcement of his death: "No flowers by special request, but if desired a memorial gift to the 'Marie Celeste' Samaritan Society of the London Hospital." A personal suggestion of this kind comes with far more force than the customary but impersonal institutional appeal, in which the endowment of a bed is put forward as the most suitable memorial. It would be interesting to learn what success has attended the late Mr. Cohen's posthumous suggestion.

### THE LIBEL ACTION AGAINST LORD BALFOUR.

The action brought by the Matron of the Clackmannan Combination Infectious Diseases Hospital, Alloa, against Lord Balfour of Burleigh for £2,000 damages in respect of an alleged libel has been dismissed in the Court of Session, Edinburgh. Miss E. B. Couper, the matron, complained of certain statements which occurred in Lord Balfour's letters to public officials concerning the death of a child, maintaining that they reflected upon her professional capacity. Three separate inquiries exonerated Miss Couper, but the defendant refused to withdraw his statements and to apologise. A judgment by Lord Dewar had previously allowed a jury trial, but the Court of Session sustained an appeal against it on the ground that the plaintiff had not relevantly averred malice. The Court, however, agreed that while an expression of regret from Lord Balfour would have been handsome and kind, a withdrawal and apology could not be demanded legally. With this crumb of comfort the plaintiff presumably will have to be content, though the sense of grievance which has led her to fight so hard for what the law denies will probably make her think it insufficient. It is a very great pity indeed that the matter was not settled out of Court, and great sympathy will be felt with Miss Couper.

### THE PUBLIC ADVERTISEMENT OF APPOINTMENTS.

On January 15 the death took place of Dr. G. G. Bantock, a veteran gynæcologist of seventy-six years, who had served many hospitals faithfully and well. He had been in retirement for some years, and was best known in connection with the Samaritan Hospital, to which he was consulting surgeon. He was also consulting surgeon to the Grosvenor Hospital for Women and the cottage hospitals at Sidcup and St. Mary Cray; and had once been resident surgeon at the Royal Maternity Hospital, Edinburgh. Dr. Bantock was a past President of the British Gynæcological Society. A reluctance to adopt the teachings of Lister prevented him from reaching the highest eminence as a surgeon, which otherwise his mastery of technique might have rendered attainable. The Samaritan Hospital is also losing the services of a surgeon of the active staff, Mr. A. G. Butler-Smythe, whose resignation has been tendered. The Middlesex Hospital requires a physician in the place of Sir James Kingston Fowler, K.C.V.O., M.D., who has reached the age limit. The name and reputation which Sir James has made for himself are well known in the institutional world, and have naturally reflected credit upon his hospital. The best known of his writings is the excellent book upon diseases of the lungs, which he wrote in conjunction with Sir R. J. Godlee. A vacancy is also declared for an assistant physician, and the joint announcement of these vacancies is worded in such a way as to make it appear that competitors are warned off as far as the senior staff vacancy is concerned. It may be that an outsider's chance of election would be so small as to be negligible, but it would be preferable not to allow an innuendo to this effect in the advertisement of the vacancy. Such a hint practically defeats the whole object of regulations as to compulsory public advertisement of appointments, and is contrary to the spirit if not to the actual letter of such clauses in hospital bye-laws.

### ST. BARTHOLOMEW'S SURVEYORSHIP.

The new Surveyor to St. Bartholomew's Hospital is Mr. H. Edmund Mathews, F.R.I.B.A., who succeeds the late Mr. E. B. I'Anson, F.R.I.B.A. Mr. Mathews, who was educated at the Merchant Taylors' School and University College, and was a frequent prizewinner at the London Architectural Association's School, is a partner with his father in a City firm. In 1906 he was elected to a Fellowship of the Royal Institute of British Architects. The institutional buildings which he has designed include the Queen Victoria Cottage Hospital at East Grinstead, which was enlarged in 1910 and contains twelve beds, a convalescent home at Clacton-on-Sea, and several public schools. It is not, however, necessary to insist too strongly on such experience where the post and responsibilities of institutional surveyor are concerned. The surveyor, of course, is engaged primarily with the estates and real property of the institution with which he is connected, and it is the financial and legal aspects of buildings and a knowledge of the Acts and bye-laws which affect property that are his main concern. Nevertheless, the more important of such posts are given to a man with architectural qualifications, and it is interesting, therefore, to summarise, however briefly, Mr. Mathew's institutional building work.

### PARISH PATIENTS IN COTTAGE HOSPITALS.

The Kingsbridge Board of Guardians, Plymouth, have decided after rather animated discussion to pay 3s. 6d. per week instead of half-a-crown for parish patients in the Kingsbridge Cottage Hospital. The Rev. J. J. Mallock, who put forward the motion which embodied this proposal, said he thought the Board would like to keep people from the workhouse, which they dreaded, and Mr. J. S. Walter Symons said that it would probably cost 4s. 6d. if they were forced into the workhouse. An opposite view was taken by Mr. Head, who said such a proposal was tantamount to a vote of censure on their own infirmary. Other speakers contended that the cottage hospital "was a very costly institution," and that the Board did not get the value of the money which was given to it. Dr. Williams Cock clinched the discussion, however, by affirming that the "economic management of the Cottage Hospital was not a matter for the Guardians to discuss, and finally that it would be most unsatisfactory for treatment which had begun in the cottage hospital to be continued in the workhouse infirmary. This latter statement sums up the statesmanlike view of the case, and convincingly rebukes the previous statement that to increase the grant to the hospital would be a criticism of the Board's institution. Dr. Cock is to be thanked for rescuing the discussion from an unworthy plane.

### A FAR-SEEING VICE-PRESIDENT AT HARTSHILL.

Mr. H. J. Johnson has been elected President of the North Staffordshire Infirmary, Hartshill, for the ensuing year, in succession to Colonel A. H. Heath. In the ordinary course the Senior Vice-President, Mr. F. J. Harrison, would have been elected, but unfortunately ill-health and a consequent absence abroad have intervened to prevent him from accepting the offer. In an interesting speech the retiring President called attention to the way in which mistaken grievances and complaints arose because certain members of the public did not understand that the infirmary was not, and consistently with its duties could not, be a hospital for incurables. He wished that his suggestion for the issue of an appeal, which had been made in the autumn, had been carried out; but he felt sure that a system of canvassing existed on paper that it required energy only to carry out. The response made to the building fund was very satisfactory, and a debt of gratitude was certainly due to Mr. Green for the district fund which he had raised to relieve distress during the strike, the surplus of which had been handed to the infirmary. Colonel Heath also warmly thanked Mr. Harrison for seeing more clearly than the committee the need for a pathological department, towards which he had given £3,000. It comes, therefore, as a matter of additional regret that the Vice-President who has done so much to push forward the need, now fully recognised in the best institutions for a pathological department, should be prevented by ill-health from accepting election to the office of President at Hartshill.

### AN AMUSING INSTANCE OF GENEROUS FOLLY.

The readiness of the public to respond to sensational appeals is equalled only, it seems, by its inability to understand their scope and requirements. An amusing instance of this was given in *The Times* this week in the form of a letter from an individual associated with the "Friends' War Victims Relief Fund," who wrote from Sofia indignantly reminding the public that the victims of the war were in no need of "astrachan jackets, nor portières, nor window curtains, nor even crimson damask tablecloths. But apart," proceeds the scandalised correspondent, "from objects more suitable for a rummage sale he (the official who receives and distributes the clothes) is deluged with articles of dress such as no Bulgarian woman will wear." The fashions of Streatham are not favoured in Sofia apparently. The common-sense warning is added that food, not clothes, is required, and that the best form in which to aid its procurement is naturally money. An English institution may very easily benefit by an English "pound-day" in its neighbourhood; we can hardly expect that Bulgaria and its neighbouring States would care for any other kind of pound but the sovereign. The semi-hysterical emotion which makes people sympathetically eager to give to those in need who are at a great distance never allows the majority to think over the best means of doing so. Folly is justified of her children.

### LARGE BEQUESTS TO GLASGOW INSTITUTIONS.

The late Mr. Edward Davis, a jeweller, of Glasgow, who died at Cheltenham, has bequeathed £40,000 to be divided among the Royal Infirmary, the Victoria Infirmary, and the Western Infirmary of the city. The importance of this gift recalls the somewhat similar sum given by the late Mr. Silver not long ago to three London hospitals.

### THE "STOCK MIXTURE" RESURRECTED.

Every practitioner in "club practice" has been compelled by force of circumstances—we refer particularly to the sweated wage at which he has been compelled to supply both advice and medicine to his contract patients—to rely very largely upon "stock" mixtures and "stock" pills in his treatment. These combinations of drugs are usually bought in bulk from wholesale chemists, and more rarely are compounded in quantity by the doctor or his dispenser on Sunday afternoons or other quiet times. By means of a couple of dozen such remedies, designed to combat most of the commoner ills, "club" dispensing is much simplified and vastly cheapened. Yet the system in itself is a bad one. It prevents that individual study and treatment which every patient has a right to expect from his doctor. It is one of those compromises which have been tolerated because they make for cheapness, irrespective of their nastiness. With the Insurance Act all this was to be changed. Dispensing was to be done by chemists alone, from prescriptions written to suit individual cases by well-paid and not over-worked doctors. Such at least was the picture drawn a few months ago. Hardly has medical benefit begun, however, than the stock mixture system has sprung to life again from the ashes of its cremation. A booklet compiled by Dr. Blundell, County Medical Officer of Health for Huntingdonshire (Cooper and Co., Huntingdon; price 6d.), has been published with the express object of providing panel doctors with a list of stock mixtures for their practice. A list of forty-nine formulæ is given, numbered for easier reference; and the worship of cheapness is inculcated by the calculation appended to each prescription of how much six, eight, or twelve ounces may be expected to cost. It may be true, as the author states, that the formulæ have been collected from the House Pharmacopœias of the teaching hospitals: and we are also willing to accept his statement that he does not wish to cramp individual prescribing. At the same time we think that is just what his booklet will do, and what in our opinion is a disastrous contingency. If panel doctors start at once to order mixture seventeen, or lotion nine, or gargle four, the bad old days of sweated club practice will be no worse than the much-vaunted era of National Insurance.

### THIS WEEK'S DRUG MARKET.

A distinct improvement in the tone of the Drug market is noticeable, and a fair business of a general character has been done. The demand for quinine has continued, and the rumours to the effect that an agreement has been made to restrict the output of cinchona bark are persistent, and the impression prevails that the agreement will be definitely announced shortly. If this should prove to be correct, it is practically certain that the price of quinine will be advanced by the makers, and in view of the fact that experienced buyers of large quantities are making purchases it might not be inadvisable for smaller buyers to follow their lead. Opium, morphine, and codeine remain unchanged. Citric acid is somewhat dearer. Cod-liver oil is not in great demand, and little business is likely to be done pending the commencement of the new fishing at about the end of this month. Essence of lemon continues to advance in price. The value of eucalyptus oil is well maintained; Balsam tolu is tending dearer. Belladonna-root is selling at rather low prices. Orris-root is dearer.

